# Predictors of 30-Day Postoperative Outcome after Elective Endovascular Abdominal Aortic Aneurysm Repair: A Tertiary Referral Center Experience

**DOI:** 10.3390/jcm12186004

**Published:** 2023-09-16

**Authors:** Maria P. Ntalouka, Petroula Nana, Alexandros Brotis, Athanasios Chatzis, Maria Mermiri, Konstantinos Stamoulis, Metaxia Bareka, Athanasios Giannoukas, Miltiadis Matsagkas, Eleni Arnaoutoglou

**Affiliations:** 1 Department of Anesthesiology, Faculty of Medicine, School of Health Sciences, University of Thessaly, Larissa University Hospital, 41110 Larissa, Greece; maria.ntalouka@icloud.com (M.P.N.); thanasischatzis94@gmail.com (A.C.); mmermiri@gmail.com (M.M.); konstaarist@windowslive.com (K.S.); barekametaxia@hotmail.com (M.B.); 2 Department of Vascular Surgery, Faculty of Medicine, School of Health Sciences, University of Thessaly, Larissa University Hospital, 41110 Larissa, Greece; petr.nana7@hotmail.com (P.N.); giannouk@med.uth.gr (A.G.); milmats@gmail.com (M.M.); 3 Department of Neurosurgery, Faculty of Medicine, School of Health Sciences, University of Thessaly, Larissa University Hospital, 41110 Larissa, Greece; alexgbrodis@yahoo.com

**Keywords:** abdominal aortic aneurysm, endovascular procedure, prognosis, complications, postoperative, acute kidney injury, biomarkers, myocardial infarction, arrhythmia, cerebrovascular accident

## Abstract

Background: We evaluated the 30-day postoperative outcome after elective endovascular aneurysm repair (EVAR) and the possible predictors for the 30-day postoperative outcome. Materials: Demographics, medical history, laboratory values, intensive care unit (ICU) admission and 30-day complications classified as major (major adverse cardiovascular events (MACEs), acute kidney injury (AKI) and death of any cause) and minor (postimplantation syndrome (PIS), postoperative delirium (POD), urinary tract infection (UTI) and technical graft failure) were documented (March 2016 to February 2019). Results: We included 322 patients. The majority were managed under general anesthesia (83%) with femoral cutdown (98.1%). Overall, 121 (37.5%) complications, mostly minor (*n* = 103, 31.9%), were recorded. In total, 11 patients (3.4%) developed MACEs, 5 (1.6%) experienced AKI and 2 (0.6%) died in the ICU. Moreover, 77 patients (23.9%) suffered from PIS, 11 from POD, 11 from UTI and 4 from technical graft failure. The multivariate logistic regression analysis revealed that aneurysm diameter (*p* = 0.01) and past smoking (*p* = 0.003) were predictors for complications. PAD was an independent predictor of MACEs (*p* = 0.003), preoperative neutrophil to lymphocyte ratio (NLR) of AKI (*p* = 0.003) and past smoking of PIS (*p* = 0.008), respectively. Conclusions: Our study showed that the 30-day morbidity after EVAR exceeded 35%. However, the majority of complications were minor, and the associated mortality was low. Aneurysm diameter and past smoking were independent predictors for postoperative outcome.

## 1. Introduction

The current recommendations suggest that endovascular aneurysm repair (EVAR) should be the preferred option for patients with abdominal aortic aneurysm (AAA) and a concomitant high perioperative risk, due to lower morbidity and mortality rates when compared with open conventional management [1,2]. Even though open repair is considered as a more durable solution with lower re-intervention rates when compared to EVAR in the long-term, it has been associated with higher morbidity and significantly higher mortality during the early follow-up period [3,4]. That being said, the reported 30-day postoperative morbidity in patients undergoing open repair of AAA may be as high as 50%, with a noticeably higher mortality when compared to EVAR (4.7–5% versus 1.3%) [3,4,5].

Over the years, a variety of factors, such as age, female sex, more complex aneurysm anatomy and the presence of cardiac, pulmonary and renal comorbidities, have been linked to higher rates of postoperative adverse events following the conventional management of AAA [6]. On the other hand, increased experience and the evolution of dedicated perioperative quality-improvement programs have ensured a more favorable outcome, in terms of morbidity and mortality, in patients undergoing either EVAR or open aneurysm repair [7]. However, patients treated with the endovascular approach should be considered a priori as high risk and, as in open repair, a variety of factors including sex, age and comorbid status may be indicative of future outcomes [8].

Hence, despite the minimally invasive nature of EVAR, a percentage of patients, ranging from 0 to 40%, may suffer from major or minor postoperative complications [9,10]. Of note, the wide range of reported morbidity following EVAR might be indicative of mis-interpreted follow-up outcomes and controversy on the definitions of the postoperative complications ensuing the EVAR [9]. As perioperative morbidity may lead to an unfavorable long-term outcome, both meticulous perioperative evaluation and increased awareness of potential predictors of adverse events may enhance a more individualized and optimal perioperative care [8]. Hence, we sought to assess the 30-day postoperative outcome after elective EVAR and to identify possible predictors for postoperative complications within 30-days after EVAR.

## 2. Materials and Methods

### 2.1. Study Cohort

A retrospective analysis of the prospectively collected data of consecutive patients treated electively with EVAR was undertaken from March 2016 to February 2019 in a single tertiary referral center. Although the patients were treated based on the European Society of Vascular Surgery (ESVS) guidelines, the final decision on repair was at the surgeon’s discretion and subject to the patient’s consent after discussion [1,11]. The existing data were recorded in such a way that subjects would be unidentifiable. This study was approved by the Institutional Review Board of Larissa University Hospital (605/14-02-2017) and was registered on clinicaltrials.org (NTC05647486).

### 2.2. Inclusion and Exclusion Criteria

Only patients managed with EVAR using standard bifurcated devices in an elective setting were included. Patients treated for ruptured, symptomatic, inflammatory or infectious AAA were excluded. Furthermore, patients managed with complex endovascular repair of the proximal landing zone, including fenestrated or branched endovascular repair, or the parallel graft technique (chimney) were considered ineligible. No patient was treated with a surgeon-modified graft. Cases needing iliac branched devices to achieve distal sealing were excluded. Distal landing to the common or external iliac artery (after internal iliac artery overstenting) was considered eligible. Additional exclusion criteria were the presence of any trauma or surgery within two months before EVAR, any autoimmune or systemic inflammatory disease and any malignancy. Of note, any patient with clinical or laboratory signs of infection before the scheduled operation was not offered an EVAR until a complete resolution of the infection was confirmed via a clinical, laboratory and/or imaging evaluation. Up to 2017, EVAR was performed using bilateral femoral cutdown. Since then, patients have been managed with either percutaneous access, using Proglide closure devices, or cutdown depending on the availability of materials, the surgeons’ preference and the patient’s anatomy.

### 2.3. Data Collection and Postoperative Surveillance

A dedicated database existed for the prospective collection of patients’ data, including demographics (age, sex), AAA diameter (after the application of a center lumen line using a dedicated software (3mensio, Medical Imaging B.V., Bilthoven, The Netherlands), comorbidities (hypertension, dyslipidemia, smoking, chronic renal failure, coronary artery disease, diabetes mellitus, chronic obstructive pulmonary disease, peripheral arterial disease (PAD) and venous thrombosis) and any medications (including antithrombotic agents and lipid-lowering agents). Preoperative (within 24 h before the operation) and postoperative (within 24 h after surgery) laboratory values were recorded, including hemoglobin, white blood cells (neutrophils, lymphocytes and neutrophil/lymphocyte ratio (NLR)), platelets, urea, creatinine and C-reactive protein (CRP) [12]. The length of hospital stay (in days) and the need for and length of stay in the intensive care unit (ICU) were also recorded and analyzed.

Early follow up with clinical, laboratory and imaging evaluation took place at the 30th day postoperatively. The imaging included computed tomography angiography with the exception of patients suffering from chronic renal disease. In these cases, a duplex ultrasonography was performed to exclude the presence of endoleaks and to estimate the sac diameter. All patients were evaluated in person at the outpatient clinic. Any adverse events, including major adverse cardiovascular events (MACEs), acute kidney injury (AKI), postimplantation syndrome (PIS), postoperative delirium (POD), urinary tract infection (UTI), technical graft failure and death of any cause, were recorded. For the purpose of this study MACEs, AKI and death were classified as major complications, while PIS, POD, UTI and graft technical failure were considered minor complications.

### 2.4. Definitions

Regarding the preoperative characteristics, hypertension was defined as the presence of a systolic or diastolic blood pressure higher than 140 mmHg or 90 mmHg, respectively, or the presence of antihypertensive medication [1]. Dyslipidemia was recorded as the presence of low-density lipoprotein cholesterol (LDL) exceeding 70 mg/dL or the use of statins [13]. Smoking was defined as any current or past, within 6 months, tobacco use, regardless of the duration of use. All patients reporting smoking cessation at least 6 months before EVAR were considered past smokers. Chronic renal failure, according to the KDIGO criteria, was taken as any glomerular filtration rate (GFR) <60 mL/h/1.73 m^2^, using the Cockcroft–Gault equation, or the need for dialysis [14,15]. Coronary artery disease included any previous myocardial infarction, percutaneous transcatheter coronary angioplasty or coronary aortic bypass [12]. Peripheral arterial disease anamnesis included all patients presenting an ankle-brachial index < 0.9 or intermittent claudication or severe atheromatosis (>70% stenosis) of the iliofemoral axis in the preoperative CTA or previous endovascular or open intervention related to lower-limb arterial disease [16].

As far as postoperative morbidity is concerned, MACEs included myocardial infarction (any clinical symptoms associated with acute coronary syndrome and/or any new electrocardiographic sign or high-sensitivity troponin elevation), arrhythmia (any event of atrial or ventricular tachycardia with more than 90 pulses per minute or any episode of bradycardia of less than 50 pulses per minute) and stroke (any transient ischemic attack, any stroke: major or minor according to Rankin Score) [12]. PIS was defined as the presence of at least two of the systemic inflammatory response syndrome (SIRS) criteria including fever >38 °C and leucocytosis >12,000/μL, without any apparent clinical or biochemical sign of infection (negative urine and blood cultures and chest radiography) [17]. AKI was defined according to the RIFLE (Risk, Injury, Failure, Loss of kidney function and End-stage kidney disease) criteria, as a twofold increase in serum creatine or >50% decrease in GFR (estimated with the Cockcroft–Gault equation) [18]. UTI refers to significant bacteriuria in a patient with symptoms or signs attributable to the urinary tract and no alternate source [19]. Of note, according to our protocol, all EVAR patients were catheterized in the bladder. Finally, POD was defined as an acute and fluctuating alteration of mental state of reduced awareness and disturbance of attention based on validated screening tools [20,21]. The Montreal cognitive assessment (MoCA) screening tool was used for the screening of POD. The evaluation was performed by a trained physician every 8 h postoperatively until hospital discharge.

### 2.5. Outcomes

The assessment of 30-day postoperative morbidity and mortality in patients undergoing elective EVAR and the identification of possible predictors for postoperative complications within 30-days after EVAR were defined as the outcomes of this study.

### 2.6. Statistical Analysis

Continuous and discrete data were presented using descriptive statistics, including means (and standard deviations) and counts (and percentages). Additionally, the role of potential modifiers was studied using the chi-square test for categorical variables or the T-test and ANOVA (or their nonparametric equivalents, the Wilcoxon and Kruskal–Wallis tests, when appropriate). Finally, a multivariate logistic regression analysis was carried out based on statistically important modifiers for each complication group to verify any actual predictive role. All statistical analyses were conducted using the statistical environment R (https://www.r-project.org/). The level of statistical significance was set at 0.05.

## 3. Results

**Figure 1:**
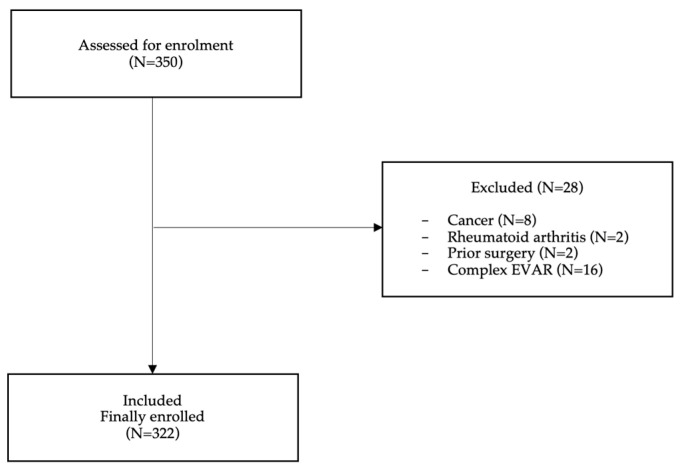
Study flowchart.

A total of 350 patients were assessed for enrollment. Of these, 28 patients were excluded: 8 patients were suffering from cancer and 2 from rheumatoid arthritis, 2 patients had undergone prior surgery and 16 patients were scheduled for complex EVAR; hence, 322 (92%) were finally enrolled (Figure 1). The mean age of the participants was 72.3 ± 7.2 years, and the mean aneurysm diameter was 59.2 ± 12.7 mm. All patients were managed with femoral cutdown, except 6 (1.9%) who were managed using closure devices. The majority of patients were males (98.1%), and most patients were operated on under general anesthesia (83%). Locoregional anesthesia was provided to the remaining cases; 4% underwent EVAR under local anesthesia. Hypertension was the most common (86.2%, *n* = 249) comorbidity, followed by dyslipidemia (78.9%, *n* = 228). The median LOH was 4 days (IQR 2 days). The majority of our patients (*n* = 228) were treated with lipid-lowering agents, mainly statins, and almost half of them (*n* = 114) were on antithrombotics. The patients’ baseline characteristics are presented in Table 1A,B.

### 3.1. Primary Outcome—Overall Postoperative Morbidity and Mortality

Overall, 121 (37.5%) complications, mostly minor (*n* = 103, 31.9%), were recorded (Table 2A). Moreover, 77 patients (23.9%) presented with PIS, 11 patients (3.4%) suffered from POD, 11 from UTI and 4 (1.2%) patients from technical graft failure. As far as major complications are concerned, fortunately, the associated morbidity was as low as 5.6% (*n* = 18). Furthermore, 11 patients (3.4%) developed MACEs, 5 patients (1.6%) experienced AKI and 2 patients (0.6%) were unfortunately admitted in the ICU, and both died: 1 due to extensive ischemic colitis after hypogastric artery occlusion and the other due to myocardial infarction. Due to the small number of events, no further analysis on death will follow (Table 2B).

### 3.2. Secondary Outcome

#### 3.2.1. Overall Complications

**Figure 2:**
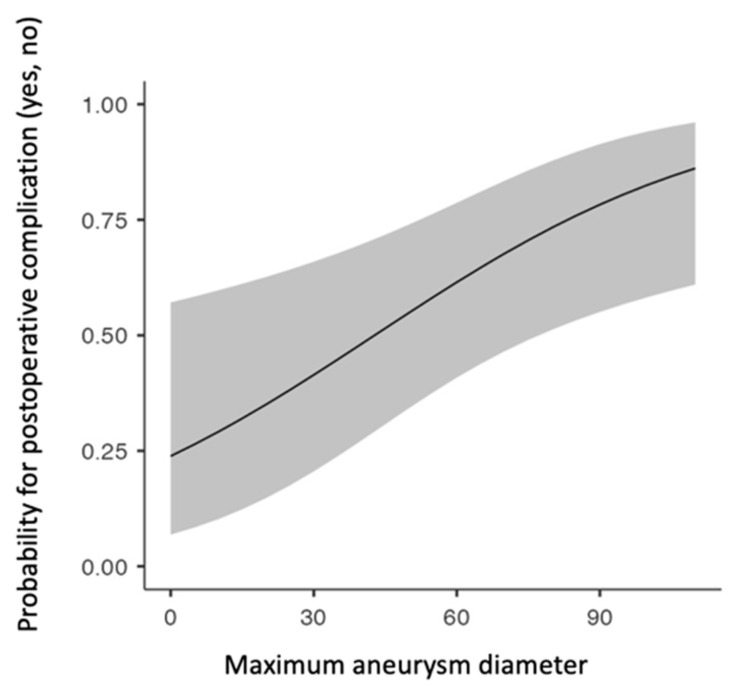
The probability for postoperative complications increases with the maximal diameter of the aneurysm in patients managed electively with EVAR.

The univariate analysis revealed that the postoperative complications, including any type of the predefined adverse events, were associated with initial aneurysm diameter (*p* = 0.016, Figure 2), past smoking status (*p* = 0.004) and PAD (*p* = 0.029). The role of maximal aneurysm diameter (OR 1.28, 95% CI 1.01–1.05, *p* = 0.01) and past smoking (OR 0.38, 95% CI 0.19–0.71, *p* = 0.003) in the development of postoperative complications was further verified using the multivariate model. Of note, no association was found between the administered pharmaceutical agents, such as the antithrombotics and the lipid-lowering factors, as well as the type of anesthesia, and the postoperative morbidity.

#### 3.2.2. Major Complications—MACEs

**Figure 3:**
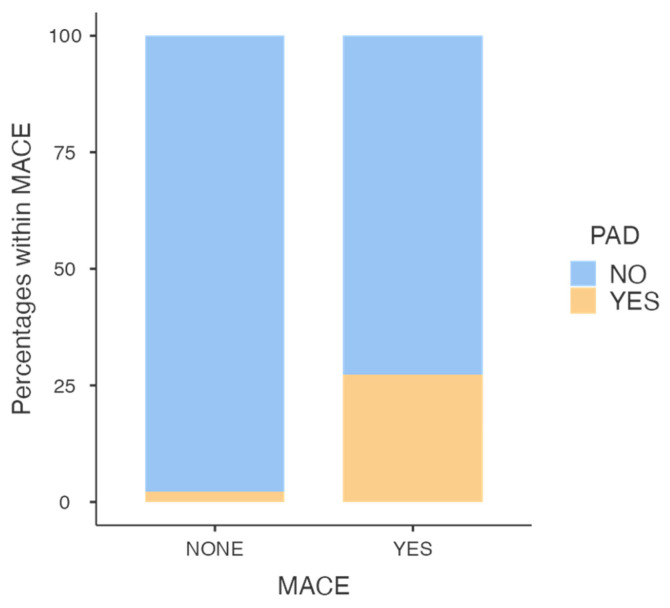
Peripheral arterial disease was an independent predictor of major cardiovascular adverse events after EVAR.

COPD (*p* = 0.032), past medical history of venous thrombosis (*p* = 0.034) and PAD (*p* < 0.001) were related to the development of MACEs. The multivariate analysis showed PAD as an independent predictor for MACEs after EVAR (OR 13.94, 95% CI 2.4–81.16, *p* = 0.003, Figure 3).

#### 3.2.3. Major Complications—AKI

**Figure 4:**
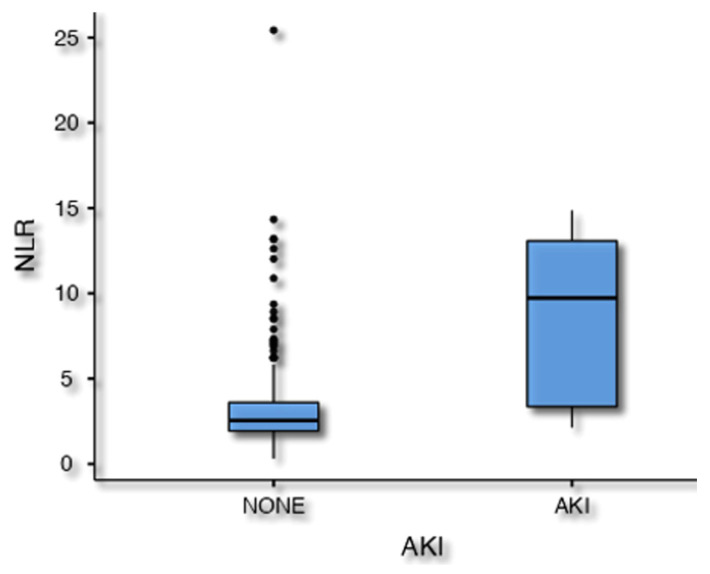
Patients with a higher preoperative neutrophil to lymphocyte ratio (NLR) (were characterized by an increased risk of developing acute kidney injury (AKI) after endovascular aneurysm repair (EVAR).

AKI was related to age (*p* = 0.024), preoperative lymphocyte titer (*p* = 0.025) and estimated NLR (*p* < 0.001) in the univariate analysis. Following the multivariate analysis, only the preoperative NLR value was still associated with AKI (OR 1.2, 95% CI 1.01–1.43, *p* = 0.003, Figure 4).

#### 3.2.4. Minor Complications

As far as the predefined minor complications are concerned, univariate analysis identified predictors only for preoperative lymphocyte titers (*p* = 0.019), and past smoking (*p* = 0.01) was associated with a higher incidence of PIS in post-EVAR patients, based on univariate analysis. The multivariate regression analysis revealed that past smoking is an independent predictor of PIS (OR 0.36, 95% CI 0.17–0.76, *p* = 0.008).

The results of the multivariate analysis are depicted in Table 3.

## 4. Discussion

This study showed that the early postoperative morbidity within 30 days following elective standard EVAR in patients treated mostly under general anesthesia exceeds 35%. Nonetheless, the vast majority of complications were minor (*n* = 103, 31.9%), and the associated mortality was estimated as null (0.6%). Nowadays, EVAR has been established as a minimally invasive and gold-standard care for high-risk patients due to the more favorable early postoperative outcome [21,22,23]. In the United States, EVAR accounts for almost 50% of all AAA repairs [23]. However, the reported rate of major or minor postoperative complications following EVAR may range from 0 to 40%; hence, our morbidity rate should not be considered surprising [9,10]. Although cumulative data have shown that the safety profile of EVAR is improving, many challenges seem poised to arise in the future, and conflicting data still exist due to the utilization of EVAR in everyday clinical practice [9]. Of note, increased postoperative morbidity has been associated with worse long-term prognosis and mortality rates following the aforementioned invasive procedure [9,10,23].

Moving on to the possible predictors of postoperative complications, multivariate logistic regression analysis revealed that aneurysm diameter and past smoking should be considered as independent risk factors for postoperative complications. Although our results support that smoking status should be considered as an independent risk factor for postoperative complications, Peterson et al. [24] found that smoking status does not affect the postoperative prognosis of patients undergoing EVAR. Nonetheless, preoperative smoking cessation has been implemented in both the European [1] and the American [25] guidelines since experts suggest that it may reduce the risk of postoperative complications.

As far as the impact of aneurysm diameter on postoperative prognosis is concerned, conflicting data still exist [8,26,27]. The aneurysm diameter has been previously identified as an indicator of adverse events or even death after EVAR [8,27]. The repair of larger aneurysms has been related to long-term adverse events, including re-intervention, rupture, mortality and loss to follow up [28,29]. The noncompliance of these patients to the postoperative surveillance potentially showed patients with less access to medical facilities or decreased ability of self-care. Under this spectrum, the large preoperative diameter might truthfully be a predictor of postoperative morbidity, as indicated by this study, as larger aneurysms may be found and treated in patients with lower compliance to medical instructions or follow up for other comorbidities.

Although our results showed that PAD should be considered as a predictor of MACEs, the past medical history of coronary artery disease was not related to early MACEs in our study sample. Patients at different risk levels were included under the definition of CAD, including patients previously managed through CABG or coronary angioplasty, and probably, this factor affected the outcomes of this analysis. However, coronary artery disease at baseline has been identified as a strong predictor for MACEs postoperatively in the long term [30]. Of note, PAD has been recognized as an important factor, maybe of higher prominence than coronary or carotid artery disease, of postoperative cardiovascular complications [31,32,33,34]. In addition, the presence of disease in multiple vascular territories has been identified as a malignant condition needing meticulous care and clinical alertness [31]. Among AAA patients, the incidence of PAD may exceed 45%, depicting that AAA patients are at high risk of diffuse atherosclerotic disease [35]. While previous analyses focused on the impact of PAD in EVAR outcomes, especially regarding re-interventions and access complications, we found that patients undergoing EVAR and suffering from PAD were at increased risk for MACEs [34,36]. This finding highlights the hypothesis that AAA patients with PAD should be considered as suffering from polyvascular disease, and more attentive pre- and intra-operative care should be provided.

AKI after EVAR presented an incidence of up to 3.0% in previous studies, while in this analysis, the estimated rate was only 1.5% even though preoperative chronic kidney disease was estimated at 8% [37]. Previous analyses have shown that preoperative NLR has been related to adverse events [12,38,39]. Especially, an elevated preoperative NLR has been associated with postoperative mortality regardless of the presence of other comorbidities [39]. Of note, our team showed in a previous study, with a smaller size sample of the same cohort, that postoperative NLR was also predictive of AKI after EVAR [12]. Similar findings were also supported by recently published data where the platelet to lymphocyte ratio was related with post-chimney-EVAR AKI [40]. These findings highlight the fact that a simple, inexpensive and routine preoperative marker might be used and evaluated as a predictor of postoperative morbidity and mortality.

Finally, PIS represented almost 25% of the total postoperative complications in this cohort. Previous studies showed a range between 11.2% and 35% [17,41,42,43]. Although a noninfectious inflammatory response after EVAR is considered a benign condition, the literature has linked PIS to major cardiovascular adverse events in the long term [44]. Multiple factors were related to the development of PIS after EVAR, including thrombus formation and stent-graft material [34,35,36]. In the current analysis, PIS was related to the preoperative smoking status—a finding not reported previously. Probably, the higher inflammatory burden of the arterial wall in previous smokers than in nonsmokers might be used as an explanatory mechanism for a more aggressive postoperative inflammatory reaction [45].

### Limitations

Despite the prospective collection of patients’ data, the retrospective nature of this study is an important limitation of this analysis. Furthermore, the limited number of patients and the short period of follow up does not permit firm conclusions. However, none of the patients was lost to the initial follow up. Most patients were male, and the findings should be taken into consideration cautiously when applied to female patients. Nevertheless, the prevalence of abdominal aortic aneurysm is significantly lower in females than in males [1]. In fact, the prevalence of abdominal aortic aneurysm is up to fourfold less in women that in men, while the pooled prevalence of abdominal aortic aneurysm in women over 60 years may be as low as 0.7% [1]. Additionally, a variety of devices were used, and some of them have been related in the past with a higher incidence of PIS. Distal landing to the external iliac artery with internal iliac artery overstenting was included even though this subgroup may be prone to an increased risk of limb occlusion [46]. Regarding the impact of access, only six patients were managed with a completely percutaneous approach, and this reflects the nascent experience of our center. Due to the small number of cases, a secondary analysis estimating the role of access type in EVAR morbidity could not be executed. Regarding baseline comorbidities, the timespan from diagnosis or treatment initiation was not available, and further analysis regarding the impact of chronicity was not provided. Hence, these confounders may have potentially affected the outcomes. The proximal neck anatomy was not analyzed and was not considered as an exclusion criterion if the patient was treated with standard EVAR. Lastly, some anatomic characteristics such as thrombus formation during the early follow up were not examined regarding their impact on postoperative morbidity and especially PIS.

## 5. Conclusions

The 30-day morbidity after elective EVAR under general anesthesia exceeded 35% in this analysis. However, the associated mortality was relatively low—less than 1%. Past smoking and AAA diameter seemed to be independent predictors of postoperative complications at 30-day follow up, while PAD and elevated preoperative NLR were independent predictors for postoperative major complications.

## Figures and Tables

**Table 1:** (**A**). Baseline characteristics. (**B**). Baseline characteristics.

(A)		
Baseline Characteristics	Mean Value	Standard Deviation (SD)
Age	72.3	7.21
Preoperative aneurysm diameter (milimeter)	59.2	12.7
Preoperative WBC	8419.3	7296.7
Preoperative neutrophils	3740.9	5480.8
Preoperative lymphocytes	1335.4	1201.7
Preoperative platelets	219.4	68.8
Preoperative platelet/lymphocyte ratio	128.0	68.9
Preoperative neutrophils/lymphocytes ratio	3.3	2.6
Preoperative hemoglobin	13.5	2.1
Preoperative creatinine	1.2	0.7
**(B)**		
**Baseline Characteristics**	**Patients (N)**	**(%)**
Males	316	98.1
Smoking	203	70.3
Current smoker	106	34.1
Past smoker	97	31.2
Never smoked	86	26.7
Not applicable	33	10.2
Hypertension	249	86.2
Coronary artery disease	138	47.8
Cerebrovascular events	24	8.3
Peripheral arterial disease	10	3.5
Chronic obstructive pulmonary disease	145	50.2
Dyslipidemia	228	78.9
Diabetes mellitus		
Type II	49	17.0
Type I	0	0
Chronic kindey disease	22	7.6
Venous thromboembolism	3	1.0
Malignancy	18	6.2

**Table 2:** (**A**). Primary outcome—overall postoperative morbidity and mortality. (**B**). Primary outcome—major and minor complications.

Postoperative Complications	N	%
**(A)**		
Overall	121	37.5
Major	18	5.6
Minor	103	31.9
**(B)**		
Major	18	5.6
MACEs *	11	3.4
AKI **	5	1.6
Death	2	0.6
Minor	103	31.9
PIS ***	77	23.9
POD ****	11	3.4
UTI *****	11	3.4
Technical graft failure	4	1.2

* MACEs: major adverse cardiovascular events; ** AKI: acute kidney injury; *** PIS: postimplantation syndrome; **** POD: postoperative delirium; ***** UTI: urinary tract infection.

**Table 3:** Multivariate regression analysis for complication development.

Morbidity	Parameter	Comparator	OR (95% CI)	*p*
Complications (*n* = 121)	Maximal aneurysm diameter	(-)	1.28 (1.01–1.05)	0.01
	PAD * (Reference level: No)	Yes	4.65 (0.87–24.7)	0.072
	Smoking status (Reference level: Never)	Current	0.84 (0.46–1.53)	0.575
		Past smoker	0.38 (0.19–0.71)	0.003
MACEs **	Venous thromboembolism		0 (0-inf)	1.000
	PAD	Yes	13.94 (2.4–81.16)	0.003
	COPD ***	Yes	4.06 (0.81–20.39)	0.086
AKI ****	Age	(-)	0.91 (0.81–1.02)	0.097
	Preoperative lymphocyte levels	(-)	0.99 (0.99–1.00)	0.136
	Preoperative NLR *****	(-)	1.2 (1.01–1.43)	0.036
PIS ******	Preoperative lymphocyte value	(-)	1.0 (1.0–1.0)	0.084
	Smoking status (Reference level: Never)	Current	1.24 (0.66–12.26)	0.519
		Past smoker	0.36 (0.17–0.76)	0.008

* PAD: peripheral arterial disease; ** MACEs: major adverse cardiovascular events; *** COPD: chronic obstructive pulmonary disease; **** AKI: acute kidney injury; ***** NLR: neutrophil to lymphocyte ratio; ****** PIS: postimplantation syndrome.

## Data Availability

The data presented in this study are available on request from the corresponding author. The data are not publicly available due to ethics and general data protection regulations.
